# Antimicrobial usage at a large teaching hospital in Lusaka, Zambia

**DOI:** 10.1371/journal.pone.0228555

**Published:** 2020-02-10

**Authors:** Anne M. Masich, Ana D. Vega, Patricia Callahan, Amber Herbert, Sombo Fwoloshi, Paul M. Zulu, Duncan Chanda, Uchizi Chola, Lloyd Mulenga, Lottie Hachaambwa, Neha S. Pandit, Emily L. Heil, Cassidy W. Claassen

**Affiliations:** 1 Virginia Commonwealth University Health System, Richmond, Virginia, United States of America; 2 Jackson Health System, Miami, Florida, United States of America; 3 University of Maryland School of Pharmacy, Baltimore, Maryland, United States of America; 4 University of Maryland School of Medicine, Baltimore, Maryland, United States of America; 5 Division of Infectious Diseases, Department of Medicine, University of Zambia School of Medicine, Lusaka, Zambia; 6 Adult Infectious Diseases Center, University Teaching Hospital, Lusaka, Zambia; 7 Department of Pharmacy, University Teaching Hospital, Lusaka, Zambia; 8 Division of Infectious Diseases, Department of Medicine, Vanderbilt University Medical Center, Nashville, Tennessee, United States of America; 9 Vanderbilt Institute for Global Health, Nashville, Tennessee, United States of America; 10 Ministry of Health, Ndeke House, Lusaka, Zambia; 11 Center for International Health, Education, and Biosecurity, University of Maryland School of Medicine, Baltimore, Maryland, United States of America; 12 Division of Infectious Diseases, Institute of Human Virology, University of Maryland School of Medicine, Baltimore, Maryland, United States of America; The University of Georgia, UNITED STATES

## Abstract

Antimicrobial resistance is a growing global health concern. Antimicrobial stewardship (AMS) curbs resistance rates by encouraging rational antimicrobial use. However, data on antimicrobial stewardship in developing countries is scarce. The objective of this study was to characterize antimicrobial use at the University Teaching Hospital (UTH) in Lusaka, Zambia as a guiding step in the development of an AMS program. This was a cross-sectional, observational study evaluating antimicrobial appropriateness and consumption in non-critically ill adult medicine patients admitted to UTH. Appropriateness was defined as a composite measure based upon daily chart review. Sixty percent (88/146) of all adult patients admitted to the general wards had at least one antimicrobial ordered and were included in this study. The most commonly treated infectious diseases were tuberculosis, pneumonia, and septicemia. Treatment of drug sensitive tuberculosis is standardized in a four-drug combination pill of rifampicin, isoniazid, pyrazinamide and ethambutol, therefore appropriateness of therapy was not further evaluated. The most common antimicrobials ordered were cefotaxime (n = 45), ceftriaxone (n = 28), and metronidazole (n = 14). Overall, 67% of antimicrobial orders were inappropriately prescribed to some extent, largely driven by incorrect dose or frequency in patients with renal dysfunction. Antimicrobial prescribing among hospitalized patients at UTH is common and there is room for optimization of a majority of antimicrobial orders. Availability of certain antimicrobials must be taken into consideration during AMS program development.

## Background

In 2015, the World Health Organization (WHO) officially recognized the growing problem of worldwide antimicrobial resistance, leading to the development of a global action plan to increase awareness and surveillance, improve antimicrobial prescribing practices, and reduce the incidence of infections.[1] Overuse and misuse of antimicrobials generates microbial selection pressure, resulting in resistance that renders existing antimicrobials ineffective.[2] As the incidence of healthcare-associated infections rises, consumption of antimicrobials increases, thereby driving further resistance.[2] Despite multiple potential structural and logistical barriers, antimicrobial stewardship (AMS) interventions should be implemented worldwide to address this problem.

AMS refers to the sum of activities, policies, and tools intended to encourage rational antimicrobial use, with the ultimate goal of improving patient outcomes and curbing antimicrobial resistance.[3,4] AMS programs also tend to result in medication cost-savings, since appropriate antimicrobial can lead toward overall decreased use, independent of the resource setting.[5–11]

While antimicrobial resistance is a global problem, AMS efforts vary widely by continent and country, and data on AMS in resource-limited settings are scarce. The 2015 international cross-sectional survey of 660 hospital AMS programs in 67 countries revealed vast differences in maturity of AMS programs by country.[5] Among the respondents (grouped by continent), Africa had the lowest percentage of national, regional, and hospital AMS standards, as well as the lowest involvement of infectious diseases physicians and pharmacists.[5] Beyond this survey, information on specific AMS interventions and antimicrobial use within Africa is limited mostly to South Africa, where several studies have demonstrated that targeted stewardship interventions can successfully decrease antimicrobial consumption and reduce drug costs, without affecting patient outcomes.[12,13]

This study was developed to address the gap in knowledge regarding antimicrobial usage in resource-limited settings to determine the need for AMS and guide development of AMS programs. The primary objective of this study was to characterize current antimicrobial use at a teaching hospital in Lusaka, Zambia. The secondary objective was to evaluate the clinical appropriateness of antimicrobials administered to patients.

## Methods

### Study setting

We conducted a cross-sectional, observational study evaluating antimicrobial consumption and appropriateness in non-critically ill adult medicine patients (≥16 years of age) admitted to the University Teaching Hospital (UTH) in Lusaka, Zambia. UTH is a 1,655-bed hospital that serves as the primary medical training and patient referral center for Zambia. A formal AMS program is currently being developed at UTH.

### Ethical approval and informed consent

This study was approved by the University of Zambia Biomedical Research Ethics Committee, the National Health Research Authority of Zambia, and the Institutional Review Board (IRB)of University of Maryland Baltimore (study no. HP-00081141). As this was a retrospective study of de-identified drug charts, informed consent was waived by the IRBs.

### Study sample

All non-critically ill patients admitted to one of the six general medicine wards, divided on the basis of sex, were evaluated for study inclusion. Logbooks containing general patient information and paper medical charts in each ward were reviewed over a one-week period in June 2018 to identify patients currently admitted with active antimicrobial orders. Patients with inaccessible drug charts were excluded from the study. Progress notes and medication administration charts were reviewed to evaluate if patients were receiving any antimicrobials. Antimicrobials for opportunistic infection prophylaxis and tuberculosis were not evaluated. Drug sensitive tuberculosis treatment is standardized in a four-drug combination pill of rifampicin, isoniazid, pyrazinamide, and ethambutol, whereas treatment of drug resistant tuberculosis is guided by sensitivity patterns, limiting the need and opportunities for stewardship for this disease state.

Antimicrobial consumption was evaluated based on pharmacy dispensing records from April to June 2018. The central and satellite pharmacies at UTH maintain “bin cards” to track the number of antimicrobial units dispensed to the general wards. The antimicrobial units are not a standardized measure and vary based on antibiotic packaging. Data from the cards was used to calculate the number of antimicrobial doses utilized per month for the most commonly used antimicrobials. By accessing both patient charts and pharmacy dispensing records, we were able to gather more complete data on total antimicrobial use. Finally, we used discharge diagnoses to identify the most common infectious diseases treated at UTH from January to June 2018.

To evaluate antibiotic appropriateness as a composite measure of correct dose, frequency, route, duration, and spectrum of coverage for each indication, Infectious Diseases Society of America (IDSA) guidelines, WHO guidelines, and local infectious diseases clinical expertise were utilized to determine all possible effective antimicrobial treatment regimens based on indication, site of infection, and antimicrobial availability (S1 Table).[6,16–18] Indications were gathered from physician clinical notes and ward logbooks which stated patients’ names and admission diagnoses. These logbooks were also used to record the day-to-day census of each ward. Appropriateness was evaluated by two clinical pharmacists and any conflicting assessments were re-evaluated by a third clinical pharmacist. Patient demographics, laboratory data, and microbiology data were also collected. Data analysis included the use of descriptive statistics to describe baseline demographics and outcomes.

## Results

**Fig 1:**
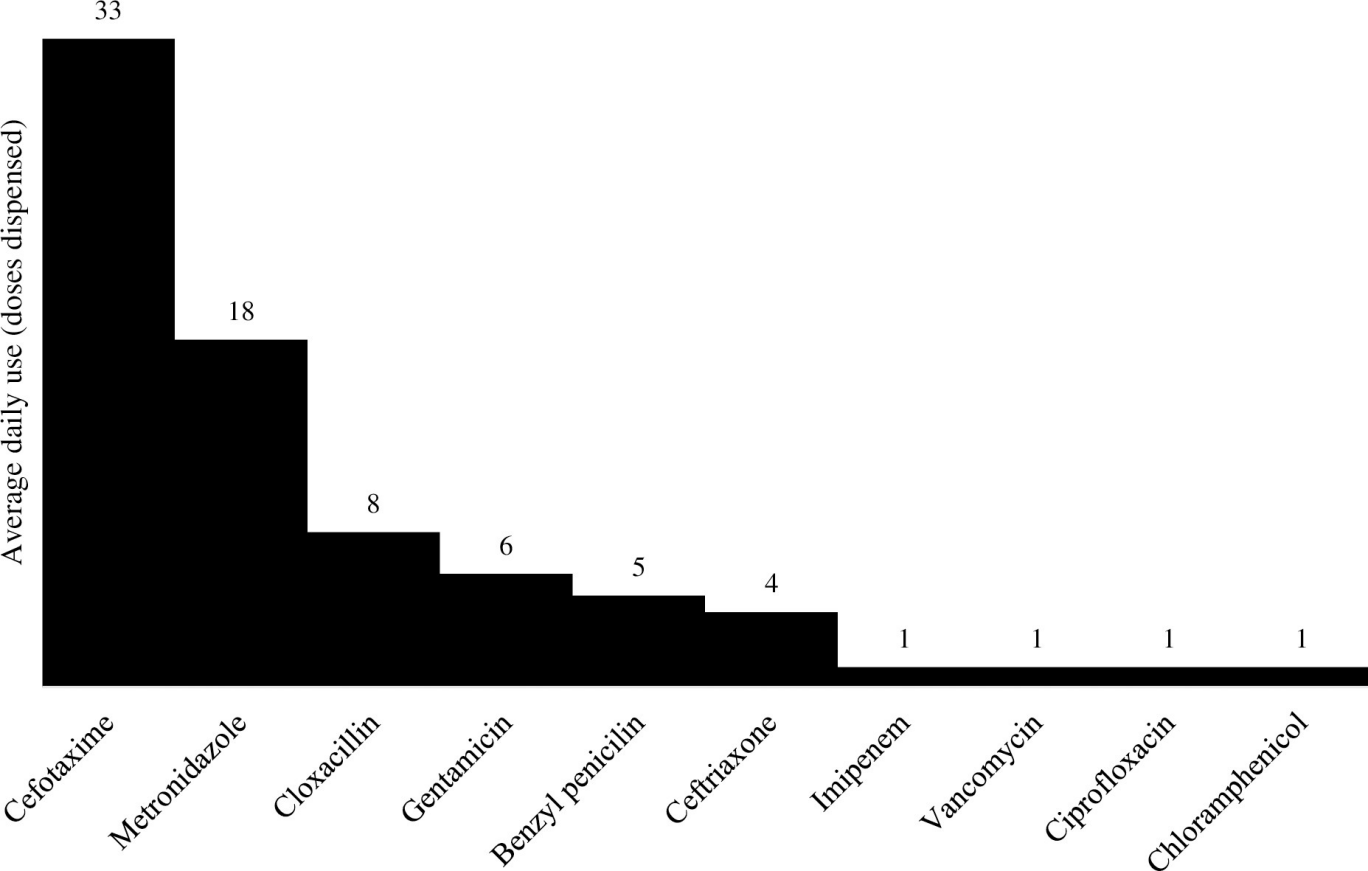
Average daily use per pharmacy dispensing records (March–May 2018)^1,2^. ^1^Average daily use = total dispensed units / typical dosing frequency per day / number of total days per three months (92). ^2^Assumed vancomycin was dosed every 24 hours.

**Fig 2:**
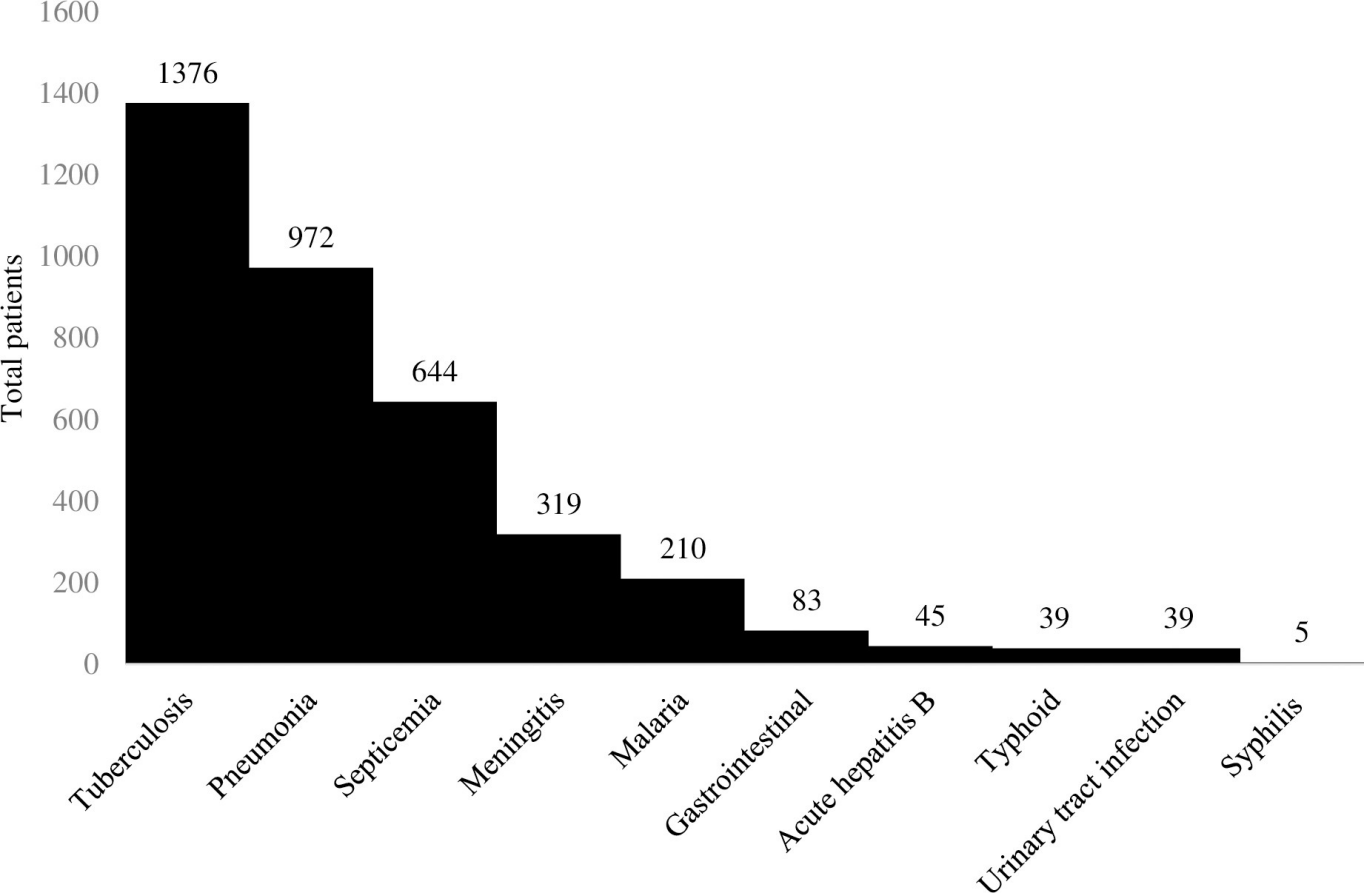
Top 10 infectious diseases treated at UTH from January to June 2018.

A total of 146 adult medicine patients were admitted to the general wards from June 8, 2018 to June 15, 2018, of which 88 (60%) patients had at least one antimicrobial ordered. Baseline characteristics of the study population are presented in Table 1. The most common antimicrobials ordered were cefotaxime (n = 45), ceftriaxone (n = 28), and metronidazole (n = 14). Thirty-five percent of patients received at least two concurrent antimicrobials. The most commonly dispensed antimicrobials over the three-month time period were cefotaxime, metronidazole, and cloxacillin (Fig 1). The most commonly treated infectious diseases at UTH were tuberculosis, pneumonia, and septicemia (Fig 2).

**Table 1:** Baseline demographics and characteristics of patients receiving antibiotics.

Characteristic	Study population (n = 88)
Age (years), median, (IQR)	41 (30–53)
Male, n (%)	46 (53)
Serum creatinine concentration (mg/dL), median (IQR)	0.87 (0.57–2.47)
Cockcroft-Gault creatinine clearance (mL/min), median (IQR)	90.9 (31.8–146.4)
White blood cell count (x10⁹/L)	9.2 ± 8.7
Cultures obtained, n (%)^a^	28 (32)
Urine	16 (18)
Blood	4 (5)
Sputum	4 (5)
Unknown	6 (7)
Organism isolated, n	
Unspeciated gram positive bacteria	6
Unspeciated gram negative rods	1
*Escherichia coli*	3
*Enterobacter spp*.	2
*Proteus spp*.	1
*Candida spp*.	4
Anti-infective ordered, n (%)	
Cefotaxime	45 (35.7)
Ceftriaxone	28 (22.2)
Metronidazole	14 (11.1)
Erythromycin	9 (7.1)
Penicillin	6 (4.8)
Amoxicillin	4 (3.2)
Azithromycin	4 (3.2)
Ciprofloxacin	4 (3.2)
Cefuroxime	2 (1.6)
Amphotericin B	1 (0.8)
Chloramphenicol	1 (0.8)
Clarithromycin	1 (0.8)
Cloxacillin	1 (0.8)
Doxycycline	1 (0.8)
Fluconazole	1 (0.8)
Gentamicin	1 (0.8)
Mebendazole	1 (0.8)
Nitrofurantoin	1 (0.8)
Tinidazole	1 (0.8)
Number of antimicrobials ordered per patient	
1	57 (64.8)
2	28 (31.8)
3	2 (2.3)
4	1 (1.1)

^a^ Two patients had both blood and urine cultures obtained.

Thirty-three percent of patients had appropriate antimicrobials ordered based on correct dose, frequency, route, duration, and spectrum of coverage for each indication (Table 2). Fifty-one percent of patients had inappropriate antimicrobial dosing and/or frequency based on renal function and/or documented indication. Intravenous antimicrobials were ordered when oral antibiotics were appropriate for 17% of patients. Appropriateness of antimicrobial treatment duration and indication could not be fully assessed due to incomplete documentation for a majority of the study population (88% duration; 57% indication). Antimicrobial double-coverage, or simultaneous orders for two antimicrobials with overlapping spectrum, was determined to be inappropriate for 12.5% of patients. On the other hand, 23% of patients did not receive adequate empiric antimicrobial coverage based on documented indications including, central nervous system infections, pneumonia, and sepsis.

**Table 2:** Antimicrobial appropriateness.

Outcome	Study population, n (%) (n = 88)
Overall inappropriate antimicrobial orders^a^	59 (67)
Dose	26 (29.5)
Frequency	26 (29.5)
Route	15 (17)
Duration	2 (2.3)
Indication	8 (9.1)
Double coverage	11 (12.5)
Insufficient coverage by indication	21 (24)
Central nervous system	9 (10)
Pneumonia	8 (9)
Septicemia	4 (5)

^a^Unable to evaluate: n = 1 (dose), n = 2 (frequency), n = 77 (duration), n = 50 (indication).

## Discussion

This study characterized current antimicrobial use in the non-critically ill male and female general medicine wards at a large Zambian teaching hospital, in order to provide guidance for developing and implementing an AMS program. We found that antimicrobial prescribing among hospitalized patients at UTH is very common and the majority of antimicrobial orders (67%) were inappropriately prescribed. Inappropriate prescribing was primarily due to wrong dose or dose frequency particularly in patients with renal dysfunction who may require lower doses or less frequent dosing intervals. Cultures, regardless of timing of initial antimicrobial administration, were obtained in only 32% of patients. Insufficient antimicrobial coverage was also common, occurring in 23.9% of patients–even in the setting of serious clinical conditions such as sepsis.

Although antimicrobial treatment selection is largely driven by the limited formulary and availability of many antimicrobials in resource-limited areas, the results of this study highlight discrepancies that exist between clinical practice, Zambian infectious diseases guidelines, and national guidelines from more developed parts of the world.[13–19] For example, the 2008 Zambian Standard Treatment Guidelines recommend using benzyl penicillin or ampicillin (plus chloramphenicol) for the treatment of meningitis in adults, while 2004 IDSA Practice Guidelines for the Management of Bacterial meningitis recommend vancomycin plus a third-generation cephalosporin.[20] Evidence is cited in the IDSA guidelines for cephalosporins–namely that they have demonstrated superiority in clinical trials against *Haemophilus influenzae* meningitis, when compared to chloramphenicol. Third-generation cephalosporins are considered first-line due to concern for the emergence of drug-resistant *Streptococcus pneumoniae*. There is no data to suggest that penicillin-resistance in these organisms is less prevalent in Africa than in other parts of the world. In fact, a recent systematic review of 144 African studies found that ampicillin resistance in *H*. *influenzae* was alarmingly high (median resistance 100% [IQR 76.6–100]) and penicillin-resistance in *Streptococcus pneumoniae*, although poorly reported, was 26.7%.[21] Unfortunately, due to limited resources and limited knowledge of microbial resistance patterns in Zambia, it is impractical for healthcare providers to apply more recently published antimicrobial guidelines from other parts of the world. Therefore, the development of updated infectious diseases guidelines in Zambia is key to optimizing antimicrobial use, standardizing treatment, and ultimately improving patient outcomes.

Our study highlights the critical need for epidemiological surveillance of microbial resistance in Africa, which could then guide clinical decision-making and AMS programs. The global implementation of AMS programs is necessary to combat the rise of multi-drug resistant (MDR) organisms and related morbidity and mortality. Limited data is available describing the characterization of antimicrobial use and AMS program implementation in resource-limited areas. More specifically, to the best of our knowledge, no literature exists regarding antimicrobial stewardship efforts in Zambia.

However, AMS efforts in South Africa have been successful. Groote Schuur Hospital, an academic teaching hospital in Cape Town, South Africa, demonstrated a 19.6% reduction in antimicrobial consumption and 35% reduction in antibiotic costs without increasing mortality or readmission rates after introducing a dedicated antibiotic prescription chart and weekly antibiotic stewardship ward rounds. A larger study described the implementation of AMS programs across 47 South African hospitals targeting appropriate culture obtainment, duration of therapy, overprescribing, and concurrent double coverage and found a significant reduction in mean antibiotic defined daily doses per 100 patient-days of 18.34 from pre-implementation to post-implementation AMS program.[12] Many emerging antimicrobial stewardship studies from South Africa include pharmacist-driven interventions that also include support from infectious disease providers and infection control personnel.[12,13,22]

Several limitations to this study exist, including the small sample size and short study duration. UTH utilizes paper medical charts which do not always contain complete and up-to-date information. Specifically, antimicrobial indication, clinical signs and symptoms of suspected infection, and anticipated duration of therapy are generally not clearly defined in patient charts, making it difficult to extrapolate all of the necessary data for this study. Once a documentation form was filled out completely, it was often removed from patient charts, making them unavailable to the medical staff. It is possible that data from some patients receiving antimicrobials were not included due to misplaced medical charts. However, this number is likely sufficiently small that it would not impact the outcomes of this study. Additionally, due to limited resources at UTH, laboratory results and certain antimicrobials were not always available, requiring patients and/or their family members to purchase antimicrobials from private pharmacies if they could afford to do so. While few cultures were obtained, susceptibilities for the speciated cultures were not collected, so presence of resistant organisms could not be evaluated. The pharmacy dispensing records provided a good estimate of antimicrobial consumption for the three-month period evaluated, however antimicrobials purchased by patients from outside pharmacies were not captured.

Based on the results of this study, antimicrobial-use guidelines will be developed by an interdisciplinary team to help guide clinicians to make the most appropriate antimicrobial decisions according to likely source of infection and individual patient characteristics (such as renal function) in accordance with antimicrobial availability and local resistance patterns. The guidelines will also emphasize obtaining appropriate cultures prior to administration of empiric antimicrobials and narrowing antimicrobial treatment based on susceptibilities. Additionally, providers will be educated on these guidelines and the importance of infection control throughout the implementation of the AMS program.

Multiple structural and logistical barriers to implementation of an AMS program at UTH remain. Utilizing paper medical charts may lead to misplaced or outdated documentation and results, making it difficult for AMS team members to identify and execute interventions. Due to limited laboratory resources, routine labs and culture data are not obtained on a routine basis and results may be delayed. Laboratory equipment should be operational and correctly calibrated as to accurately identify organisms and resistance patterns. Availability of certain antimicrobials (i.e. vancomycin and cefepime) fluctuates in the hospital, or may not be available at all in Zambia, and therefore, must be taken into consideration during AMS guideline development. Some antimicrobials are not part of the essential medication list and will not be supplied by the government. Following the implementation of an AMS program at UTH, future research includes post-implementation AMS program antimicrobial use, patient outcomes, and impact on resistance patterns.

Implementation of AMS programs are essential to reducing emergence of MDR organisms and improving patient outcomes. These results offer insight into antimicrobial use in a resource-limited setting currently lacking an AMS program.

## Supporting information

S1 Table Appropriate antimicrobial regimen according to site of infection. (DOCX)

S1 Dataset (XLSX)
