# Supplementary material for: Antimicrobial usage at a large teaching hospital in Lusaka, Zambia
Source: PLoS One. 2020 Feb 10;15(2):e0228555. doi: 10.1371/journal.pone.0228555 (PMC7010251; doi:10.1371/journal.pone.0228555)
Supplement: S1 Table — (DOCX) [file pone.0228555.s001.docx]

**Supplementary data**

**Table 1. Appropriate antimicrobial regimen according to site of infection**

| Site of Infection | Antimicrobials regimens considered appropriate^a^ |
| --- | --- |
| Meningitis | Ceftriaxone 2g IV every 12 hours  Cefotaxime 2g IV every 4 hours  Benzyl penicillin 4 million units IV every 4 hours (if concern for neurosyphilis)  Doxycycline 100mg PO twice daily (if concern for neurosyphilis and penicillin-allergy) |
| Pneumonia | Benzyl penicillin 1-2 million units IV every 6 hours  Amoxicillin 500mg - 1g PO every 8 hours  Ceftriaxone 1g IV once daily  Cefotaxime 1-2g IV every 8 hours  Cefuroxime 500mg PO twice daily or 1.5g IV every 8 hours  Metronidazole 500mg PO every 8 hours (if concern for aspiration)  Azithromycin 500mg PO daily OR 500mg PO once followed by 250mg PO daily  Erythromycin^b^ 500mg PO every 6 hours  Doxycycline^b^ 100mg PO twice daily |
| Urinary Tract Infection | Ceftriaxone 1g IV once daily  Cefotaxime 1-2g IV every 8 hours  Ciprofloxacin 500mg PO twice daily  Doxycycline 100mg PO twice daily |
| Gastrointestinal | Ceftriaxone 1g IV once daily  Cefotaxime 1-2g IV every 8 hours  Metronidazole 500mg PO every 8 hours |
| Sepsis | Ceftriaxone 1g IV PO daily^c^ |

Abbreviations: IV, intravenous; PO, by mouth

^a^Dosing based on normal renal function (creatinine clearance >50 mL/min). Renal dosing adjustments evaluated according to individual drug product labels.

^b^Macrolide or tetracycline monotherapy were not considered sufficient due to rising pneumococcal resistance rates.

^c^Although not considered optimal coverage (due to lack of resistant nosocomial pathogens), was considered appropriate for the purposes of this study given limited broad-spectrum agents.
